# Impact of Temperature and Soil Moisture on the Life Cycle of the Strawberry Pest *Priophorus fulvostigmatus* and Its Control

**DOI:** 10.3390/insects16070717

**Published:** 2025-07-12

**Authors:** Juan Cui, Jingxu Yin, Lihuan Dong, Yu Gao, Shusen Shi, Jingzhu Zou, Wenbo Li, Yu Wang

**Affiliations:** 1 College of Agriculture, Jilin Agriculture Science and Technology College, Jilin 132101, China; cuijuanjilin@jlnku.edu.cn (J.C.); yinjingxu20040508@163.com (J.Y.); 2 Huanren County Agricultural Comprehensive Service Center, Huanren 117200, China; dlhsh-521@163.com; 3 College of Plant Protection, Jilin Agricultural University, Changchun 130118, China; gaoy1101@163.com (Y.G.); sss-63@263.net (S.S.); 4 Key Laboratory of Soybean Disease and Pest Control, Ministry of Agriculture and Rural Affairs, Changchun 130118, China; 5 Liaoyuan City Farmer Science and Technology Education Center, Liaoyuan 136200, China; 17684388307@163.com; 6 College of Biological Resource and Food Engineering, Qujing Normal University, Qujing 655011, China

**Keywords:** *Priophorus fulvostigmatus*, temperature, soil water content, survival rate

## Abstract

*Priophorus fulvostigmatus*, a species of leaf-cutter wasp, is an important leaf-feeding pest on strawberries. We investigated the effects of temperature and soil moisture content on key life cycle parameters of *P. fulvostigmatus*. The development time, survival, fecundity, and life table parameters of *P. fulvostigmatus* were observed at five temperatures. Pupal development and survival under five moisture contents (8%, 12%, 16%, 20%, and 24%) and four durations of water immersion (0, 1, 3, and 5 d) were recorded. *P. fulvostigmatus* could complete its life cycle at a constant temperature range of 16–28 °C. The duration of the immature stage first decreased and then increased with rising temperature, being longest at 16 °C and shortest at 25 °C. Female longevity and female fecundity did not differ between the temperature range of 16–25 °C. However, survival rates at all developmental stages decreased with increasing temperature. At 28 °C, both fecundity and survival rates of *P. fulvostigmatus* were significantly reduced compared to other temperatures. Compared with that at the constant temperature of 22 °C, the developmental duration of each stage was similar at a fluctuating temperature of 22 °C. The number of eggs laid per female, the longevity of male adults, and the eclosion rate were all significantly reduced. The net reproductive rate (*R*_0_) under constant temperature conditions was significantly higher than under fluctuating temperature conditions, and the mean generation period (*T*), intrinsic rate of increase (*r*), and finite rate of increase (*λ*) differed significantly. The soil moisture content significantly impacted the pupation and eclosion of *P. fulvostigmatus*. Differences in soil moisture content had no significant effect on the duration of development; a moisture content of 8–16% was more suitable for their pupation and eclosion. Pupal development differed significantly between different periods of water immersion after the mature larvae were immersed in the soil. The longer the larvae remained in the soil, the lower their emergence rate after immersion. Thus, environmental temperature affected the growth, reproduction, and survival of *P. fulvostigmatus*. The optimal soil moisture for pupation of mature larvae was 12% to 16%. After the larvae were immersed in soil, the emergence rate was significantly reduced. These findings expand our understanding of the biological characteristics of *P. fulvostigmatus* and can facilitate the development of prevention and control strategies.

## Simple Summary

*Priophorus fulvostigmatus* is an important pest of strawberries. The effects of temperature and soil moisture on the growth, reproduction, survival, and life cycle parameters of *P. fulvostigmatus* were recorded using automatic temperature control and artificial soil moisture control in the laboratory. *P. fulvostigmatus* could complete its life cycle at a temperature range of 16 to 28 °C. However, 28 °C was unfavorable for its survival and reproduction. The fecundity and emergence rate of *P. fulvostigmatus* were lower at a fluctuating temperature of 22 °C than at a constant temperature of 22 °C. Favorable eclosion from the soil was observed for *P. fulvostigmatus* within a soil moisture content range of 12% to 16%. However, excessive soil moisture, leading to waterlogging, reduced the larval survival rates. This study enhances our understanding of the biological characteristics of *P. fulvostigmatus* and provides a basis for the prediction, prevention, and control of this pest.

## 1. Introduction

*Priophorus fulvostigmatus* has emerged as a serious pest of strawberries in parts of China, causing severe defoliation and yield loss. However, information on how environmental factors affect population dynamics is scarce, making it difficult to design effective control strategies. Overwintering adults appear in early May. Adults can mate and oviposit on the same day they emerge from their pupa. Female adults typically select strawberry petioles with optimal growth for oviposition, targeting the petioles of the second leaf nearest to the youngest leaf. The larvae have six instar stages, with food consumption increasing significantly at each stage [1]. After maturation, the larva transforms into a pupa and conceals itself in the soil. The pest was first reported on strawberries in the greenhouse of Jilin University of Agricultural Sciences in 2015, and by May 2020, it had infested strawberry fields on a large scale, with a damage rate of 100%. Young larvae feed on the mesophyll tissue from the underside of the leaves, leaving behind only the thin, transparent epidermis. Larvae in the third instar and beyond consume significantly more foliage, creating holes or notches in the leaves. In severe cases, the leaves are completely eaten, posing a serious threat to strawberry production.

The development of insects is closely related to meteorological factors, and their combined effect determines the growth, development, reproduction, and distribution of insects, as well as general ecological characteristics [2,3]. Meteorological factors include temperature, water (precipitation and humidity), and sunlight. Insects can complete their growth and development within an optimal temperature range, and their growth rate is directly proportional to the increase in temperature. However, in the natural environment, temperatures fluctuate throughout the diurnal cycle. Consequently, insects demonstrate notable variations in their life and physiological characteristics under constant and fluctuating temperature conditions [4]. In the same region, the fluctuations in water-related factors, such as precipitation and humidity, differ significantly from changes in temperature. Precipitation and drought affect the dynamics of pest populations to some extent, with a strong correlation between the frequency and intensity of pest outbreaks [5,6].

Many insects live on the ground as larvae and adults, but live underground as pupae. Compared with the aboveground stage of their life cycle, when these insects pupate in the soil, the soil moisture conditions can affect the pupation and eclosion of the insects, resulting in a marked fluctuation in the population size of each generation [7,8]. Usually, pupal mortality increases with increasing moisture content in soil [9]. Different soil moisture contents significantly affect the eclosion of *Helicoverpa armigera*, and a relative soil moisture content below 20% is favorable to the eclosion. At a relative soil moisture content greater than 40%, the eclosion reduces significantly [8,10]. Flooding detrimentally affects the pupation of *Spodoptera frugiperda* and reproduction of its adults [11]. Therefore, controlling soil moisture in the fields, for instance, using artificial rain or irrigation, can effectively prevent and control the occurrence of pests [12,13].

Accurately ascertaining the biological characteristics of pests is the basis for other related research and for targeted research and development of prediction and control techniques [14]. The larvae are mainly pests but also account for a large part of the life history of *P. fulvostigmatus*, so the influence of important ecological factors on the larval stage is more prominent. However, comprehensive and systematic research on the effects of temperature and soil moisture content on larval populations is lacking. Therefore, in this study, we aimed to systematically investigate the developmental history, survival rate, female fecundity, and life table parameters of the individual developmental stages of *P. fulvostigmatus* using strawberries as the host plant under controlled indoor and fluctuating temperature conditions. We simulated different soil humidity levels and rainfall at different times to observe the pupation and eclosion of *P. fulvostigmatus*. It is of theoretical and practical significance to understand the occurrence regularity and adaptability of the pest to the environment, improve its prediction, and determine the appropriate period for its prevention and control.

## 2. Materials and Methods

### 2.1. Rearing Insects

*P. fulvostigmatus* samples were collected from the strawberry experimental field of Jilin Agricultural Science and Technology University, Jiuzhan Campus (43°57′01″ N, 126°28′38″ E). After collection, strawberry seedlings were used as the host plants for feeding the pests in an artificial climate chamber (temperature 25 ± 1 °C, relative humidity 65% ± 5%, photoperiod 16 L:8 D). Strawberry seedlings were planted in a greenhouse without any pesticides during the growth period, and other pests were manually removed. The plants were allowed to reach a height of 10 to 15 cm for testing and were placed in an insect cage (length × width × height = 50 cm × 50 cm × 50 cm); subsequently, the larvae of *P. fulvostigmatus* were carefully directed onto the strawberries in the cage with a brush for rearing.

### 2.2. Observations and Measurements of Growth, Development, and Reproduction Indicators Under Constant and Fluctuating Temperature Conditions

*P. fulvostigmatus* typically appears in Jilin City at the beginning of May, and the infestation time is mainly between May and September. In this region, the average temperature in May, June, July, August, and September ranges from 8 to 22 °C, 15 to 26 °C, 19 to 29 °C, 17 to 27 °C, and 10 to 24 °C, respectively. We evaluated the effect of temperature variation on the life cycle of *P. fulvostigmatus* using two temperature regimes in artificial climatic chambers (PQX-450H, Zhongyi Guoke (Beijing) Technology Co., Ltd., Beijing, China) in the suitable season for the growth and development of *P. fulvostigmatus*. Preliminary tests showed that the pest could not complete its life cycle at a constant temperature of 31 °C. Therefore, according to the daily temperature fluctuation pattern in the field, the laboratory rearing condition (22 °C) was set as the control for all treatments. The constant temperature was set to five levels (16, 19, 22, 25, and 28 °C). The fluctuating temperature sequence ranged from 16 to 28 °C (Table 1). The experiment comprised 12 temperature cycles, each for 2 h. The average daily temperature obtained using this method was 22 °C. The relative humidity was 65 ± 5% with a photoperiod of 16 L:8 D across all treatments.

Strawberry petioles with eggs within 24 h were placed in plastic culture dishes (10.0 cm diameter) lined with moisturizing filter paper at the bottom. Each dish contained 3 to 4 petioles with approximately 20 eggs. The culture dishes were sprayed with water daily to keep the filter paper moist, and egg hatching was regularly observed and recorded at 8:00 and 20:00. One hundred eggs per treatment group were observed. After hatching, visibly healthy larvae were individually numbered and transferred into plastic culture dishes (10 cm diameter) and provided with fresh strawberry leaves as the food source. Sixty larvae were observed per temperature treatment, using single larvae as a replicate. Daily observations were conducted, and the molting and mortality of the larvae were recorded while promptly replacing fresh strawberry leaves as needed. After the maturation of larvae, moist and sterilized soil was prepared for pupation. The time and number of larvae entering the soil and the time and number of adult emergences were recorded. A single male and female *P. fulvostigmatus*, newly emerged, were selected from the experiment and placed in a transparent plastic container (14 cm height, 9 cm top diameter, 10 cm base diameter). Adults were provided with water through individual saturated cotton balls, and fresh strawberry plants served as the substrate for oviposition. Strawberry seedlings were replaced daily, and eggs on strawberry petioles and leaves were carefully observed under a stereomicroscope. The number of mating pairs used per treatment varied (7 to 22) depending on the number of individuals surviving to adulthood (the sex ratio was 1:1) in the developmental phenology study. The number of eggs produced by female adults and the longevity of both male and female adults from the day they started laying eggs were recorded (when eggs were first found on strawberry petioles). Males that died during trials (occurring in 15% of pairs) were replaced within 24 h. This replacement caused no observable delay in the oviposition.

### 2.3. Effects of Soil Moisture on Pupal Development and Eclosion

In the strawberry field, the sandy loam soil without pesticides and contaminants was selected, brought back to the laboratory to dry naturally, and filtered through a 40-mesh sieve. The sieved soil was placed in an electric drying oven (GZX-9023MBE, Shanghai Boxun Medical Biological Instrument Co., Ltd., Shanghai, China) and baked at a constant temperature of 105 °C until reaching a constant weight. It was then set aside. According to the configuration method described by Han et al. [15], different soil moisture contents were configured using the weighing method. A total of 100 g of dried soil was weighed, and 8 mL, 12 mL, 16 mL, 20 mL, and 24 mL of distilled water were added to prepare the soil with a natural moisture content of 8%, 12%, 16%, 20%, and 24%, respectively. Visually healthy mature larvae of the same size were selected and placed into plastic tubes (2.7 cm diameter, 10 cm height) with soils under different moisture conditions. Each tube had one larva, and each treatment was repeated 30 times. The test was conducted in an artificial climate chamber, and the upper part of the plastic tube was covered with a wet towel to reduce the evaporation of water and maintain the stability of the soil moisture content. Water was added to maintain appropriate moisture content after weighing regularly daily, and the emergence was recorded. The larval penetration rate was calculated as (number of larvae into the soil/total number of tested larvae) × 100%, and the eclosion rate as (number of adults emerged/total number of tested penetrated larvae) × 100%.

### 2.4. Effects of Soil Saturation on Development During the Pupal Stage and Eclosion

According to the results of the test described in Section 2.3, soil that was 2 cm deep with a moisture content of 16% was added to the plastic box (10 cm diameter, 6 cm height). Visually healthy mature larvae of the same size were selected and placed in the plastic box. According to the time of the mature larvae entering the soil, the water immersion treatment was conducted at 12 h, 24 h, 72 h, and 120 h (simulated rainfall in different periods). The amount of water added to the soil exceeded the saturation state and was maintained for 8 h. Each treatment was repeated 30 times. We added dry soil to the plastic box such that the soil moisture content was 16% to prevent the disturbance of larvae or pupae. After sealing with a plastic film, the film was pierced with holes. The soil moisturizing method and test conditions were the same as those described in Section 2.3. The number of eclosed adults was observed and recorded daily.

### 2.5. Statistical Analysis

Biological parameters of different constant temperature and soil moisture levels were analyzed using Tukey’s honestly significant difference (HSD/0 test in IBM SPSS Statistics (v27.0) statistical analysis software. The biological parameters at a constant temperature of 22 °C and a fluctuating temperature of 22 °C were analyzed using an independent samples *t*-test. All graphs were plotted using GraphPad Prism (v10.0). The net reproductive rate (*R*_0_), intrinsic rate of increase (*r*), mean generation period (*T*), and finite rate of increase (*λ*) were analyzed using TWOSEX-MS Chart (Ver. 5/7/2024) [16].

(1) $$R_{0}=\sum_{x=0}^{\infty }l_{x}m_{x}$$

(2) $$\sum_{x=0}^{\infty }e^{-r(x+1)}l_{x}m_{x}=1$$

*λ* = *e^r^* (3)

(4) $$T=\frac{\ln(R_{0})}{r}$$

where *l_x_* represents the survival probability from age 0 to *x*, and *m_x_* represents the average number of eggs an individual produces at age *x.*

All variances and standard errors were obtained with a bootstrap of 100,000 random samples, and differences between different treatments for each parameter were evaluated using a paired bootstrap test [17,18].

## 3. Results

### 3.1. Developmental Duration of P. fulvostigmatus at Different Temperatures

Differences in the developmental duration at various life stages of *P. fulvostigmatus* were observed under five different constant temperature conditions, as shown in Table 2. The immature stage of *P. fulvostigmatus* initially decreased and then increased with rising temperatures within a constant temperature range of 16 to 28 °C. The duration of the egg stage decreased with an increase in temperature. The average developmental period of the larval, prepupal, and pupal stages initially decreased and then increased with temperature.

**Figure 1:**
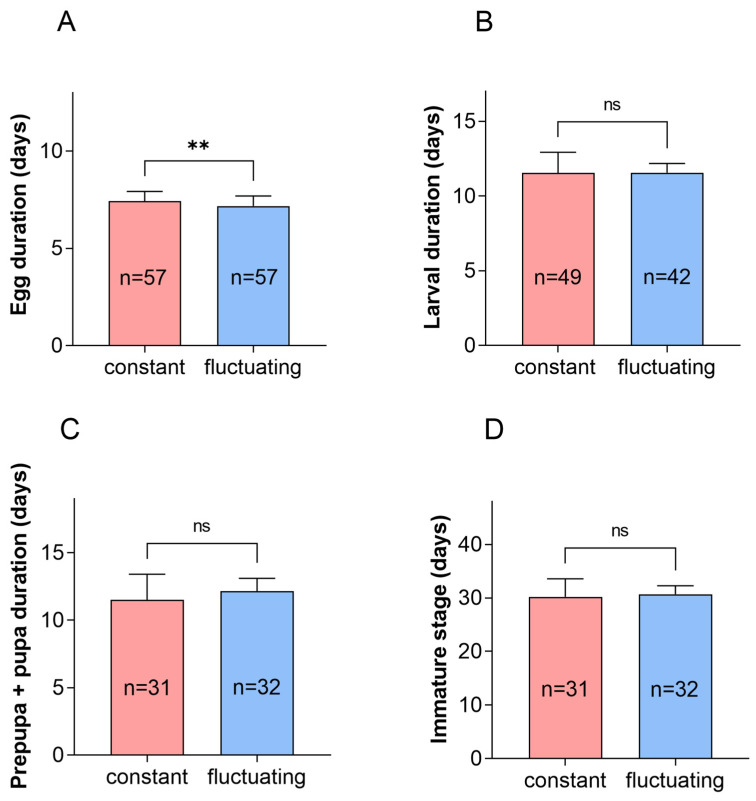
Duration of egg, larva, prepupa + pupa, and immature stages of *P. fulvostigmatus* at a constant temperature or fluctuating temperature of 22 °C. (**A**) Egg duration. (**B**) Larva duration. (**C**) Prepupa + pupa duration. (**D**) Immature stage. The data in the figure denote mean ± SE. Asterisks indicate significant differences between constant temperature and fluctuating temperature according to an independent samples *t*-test. **, *p* < 0.001; ns, not significant.

The egg stage at a fluctuating temperature of 22 °C was significantly shorter than at a constant temperature of 22 °C (Figure 1, *p* = 0.009). The duration of the larval, prepupal, pupal, and immature stages at 22 °C did not differ between fluctuating temperature conditions and a constant temperature of 22 °C.

### 3.2. Longevity and Reproduction of P. fulvostigmatus at Different Temperatures

Observational results indicate that under controlled temperature conditions (16 to 28 °C), the pre-oviposition period of female adults ranged from 0.83 to 1.66 days. Individual females over the course of a lifetime laid the most eggs (65.80 eggs) at 22 °C, which was significantly higher than that at 28 °C (*p* < 0.001), showing no significant difference compared with other treatments. The female adult lifespan was shortest at 28 °C, significantly shorter compared with other treatments (*p* < 0.001). The male adult lifespan was the shortest at 28 °C, only 2.5 days, significantly lower compared with other treatments (*p* < 0.001) (Table 3).

**Figure 2:**
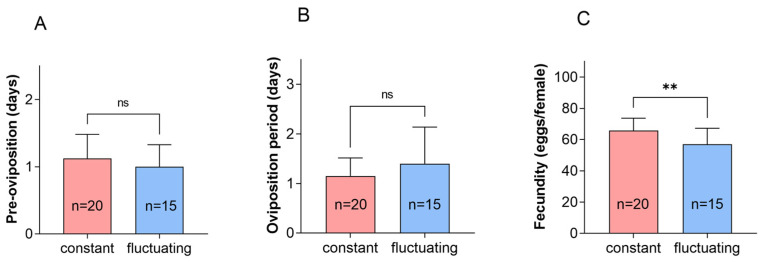
Pre-oviposition duration, oviposition duration, and fecundity of *P*. *fulvostigmatus* at a constant temperature or fluctuating temperature of 22 °C. (**A**) Pre-oviposition duration. (**B**) Oviposition duration. (**C**) Fecundity. The data in the figure denote mean ± SE. Asterisks indicate significant differences between constant temperature and fluctuating temperature based on an independent samples *t*-test. **, *p* < 0.001; ns, not significant.

**Figure 3:**
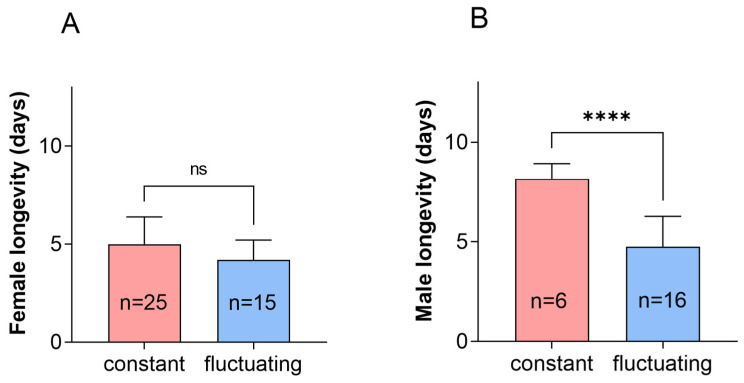
Longevity of *P. fulvostigmatus* females and males at a constant temperature or fluctuating temperature of 22 °C. (**A**) Female longevity. (**B**) Male longevity. The data in the figure denote mean ± SE. Asterisks indicate significant differences between constant temperature and fluctuating temperature based on an independent samples *t*-test. ****, *p* < 0.0001; ns, not significant.

The pre-oviposition and oviposition periods did not differ under the constant temperature of 22 °C and the fluctuating temperature of 22 °C. Under constant temperature conditions, the oviposition of adults was higher than that under fluctuating temperature conditions (Figure 2, *p* = 0.007). The lifespan of female adults did not differ, while that of male adults was significantly higher compared to that at fluctuating temperatures (Figure 3, *p* < 0.0001).

### 3.3. Egg Hatching, Larval Survival, and Adult Eclosion Rates at Different Temperatures

At a constant temperature of 16 to 28 °C, the highest hatching rate of eggs was 91.88%, and the lowest was 81.25% (*p* < 0.001). The larval survival rate was the highest at 22 °C, reaching 70.93%, which was significantly higher than that at other temperatures (*p* < 0.001). The adult emergence rate decreased with increasing temperature, with the highest at 16 °C and the lowest at 28 °C (*p* < 0.001) (Table 4).

**Figure 4:**
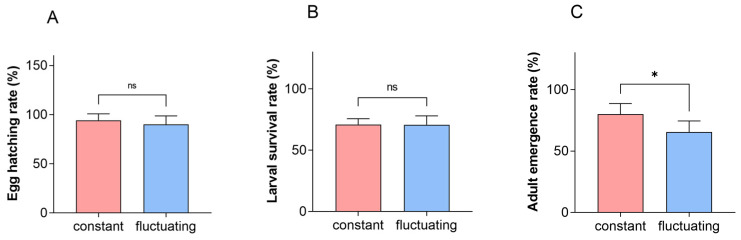
Egg hatching rate, larval survival rate, and adult emergence rate of *P. fulvostigmatus* at a constant temperature or fluctuating temperature of 22 °C. (**A**) Egg hatching rate. (**B**) Larval survival rate from first-instar to last-instar. (**C**) Adult emergence rate. The data in the figure denote mean ± SE. Asterisks indicate significant differences between constant and fluctuating temperatures based on an independent samples *t*-test. *, *p* < 0.05; ns, not significant.

There was no significant difference in the hatching rate and larval survival rate between a constant temperature of 22 °C and a fluctuating temperature of 22 °C. The adult emergence rate under fluctuating temperature conditions was significantly lower than that under constant temperature conditions (Figure 4, *p* < 0.05).

### 3.4. Population Parameters at Different Temperatures

The population parameters for *P. fulvostigmatus* are presented in Table 5. The *R*_0_ decreased with an increase in temperature and was the highest at 16 °C, significantly higher than other treatments, and 6.45 times higher than that at 28 °C. There was no significant difference at 19, 22, and 25 °C. The *T* was highest at 16 °C and significantly higher than other treatments. The *r* and *λ* were the highest at 16 °C, significantly higher than all corresponding treatment estimates except for those at 19 and 22 °C. The *r* and *λ* were the lowest at 28 °C but not significantly different from the corresponding estimates at 25 °C.

**Figure 5:**
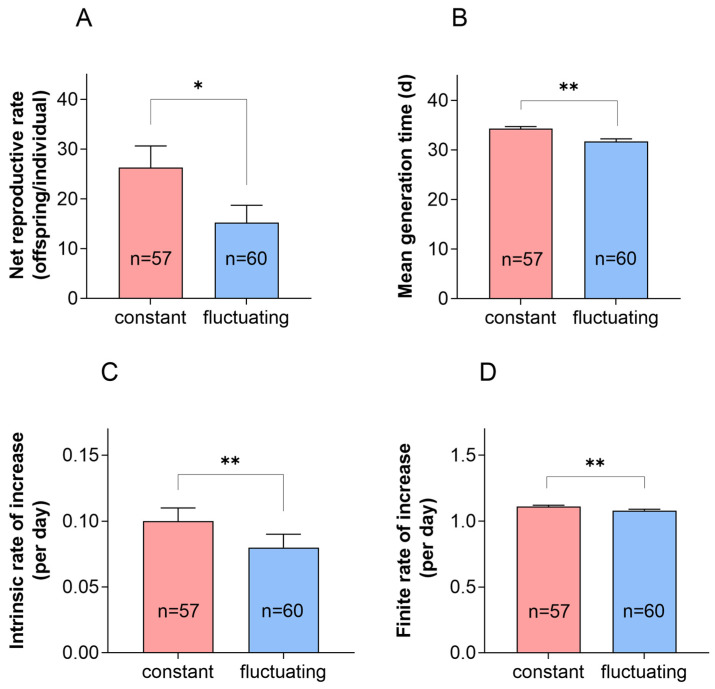
Population parameters in *P. fulvostigmatus* at a constant temperature or fluctuating temperature of 22 °C. (**A**) Net reproductive rate *R*_0_. (**B**) Mean generation time *T*. (**C**) Intrinsic rate of increase *r*. (**D**) Finite rate of increase *λ*. The data in the figure denote mean ± SE. Asterisks indicate significant differences between the constant temperature and fluctuating temperature based on an independent samples *t*-test. *, *p* < 0.05; **, *p* < 0.01.

There were significant differences in the *R*_0_, *T*, *r*, and *λ* between the variable temperature of 22 °C and the constant temperature of 22 °C (Figure 5). The *R*_0_ was significantly higher under constant temperature conditions than under fluctuating temperature conditions, and there were significant differences in *T*, *r*, and *λ*.

### 3.5. Effect of Different Soil Moisture Conditions on Pupation and Eclosion

Soil moisture significantly affected the penetration rate of mature larvae (*p* < 0.001; Table 6). When the soil moisture content was between 8% and 16%, the penetration rate of mature larvae was more than 90%. *P*. *fulvostigmatus* ceases to burrow into the soil for pupation when the soil moisture content reaches 24%. Different soil moisture contents had no significant effect on the duration of pupal development in the soil. The emergence rate of adults with a soil moisture content of 12% to 16% was more than 80%, significantly higher than that at other treatment conditions (*p* < 0.001). When the moisture content was 8% and 20%, the emergence rate was less than 60%. When the moisture content reached 24%, no *P*. *fulvostigmatus* could emerge and died.

### 3.6. Effect of Water Immersion on Pupal Development and Eclosion

There was a significant difference in pupal duration between different periods of water immersion after the mature larvae were immersed in the soil (Table 7). The longest pupal developmental period was 13.17 days in the 120 h immersion group, significantly higher than that in the 24 h immersion group (*p* = 0.037). However, no significant differences were observed compared to the other treatments. The longer the larvae remained in the soil, the lower the eclosion rate after immersion. The eclosion rate after 12 h of immersion was significantly higher than other treatments (*p* < 0.001) and 36.67% higher than that of the 120 h immersion group.

## 4. Discussion

The results of the present study showed that, in the temperature range of 16–28 °C, with an increase in temperature, the developmental period of the eggs of *P*. *fulvostigmatus* was significantly shortened. However, at 28 °C, the developmental period of the larvae, pupae, and eggs to the adult was all slightly prolonged compared with that at 25 °C, suggesting that high temperatures inhibited the metabolism of *P*. *fulvostigmatus*, consequently impacting their growth and development. Temperature is a critical ecological factor that influences the developmental progress of insects under meteorological conditions [19,20]. Within the appropriate temperature interval, the development rate of insects accelerates with an increase in temperature. However, growth and development can be restricted by both excessively high and low temperatures [21].

Under constant temperature conditions, the survival rate of *P*. *fulvostigmatus* in the high-temperature group was lower than that in the low-temperature group, similar to the results of previous studies on *Spodoptera frugiperda* [22] and *Cephlca kunyushanica* [3]. At 28 °C, eggs, larvae, and pupae all showed high mortality. Eggs are more tolerant of temperature changes than larvae and pupae. Differences in the temperature tolerance of insects in different insect states have been reported in *Xestia c-nigrum* [23] and *Mesoneura rufonota* [24]. Exposure to high temperatures causes water loss in insect bodies, shortened longevity, and decreased fecundity [25]. We found that the oviposition of females was the lowest at 28 °C, indicating that high temperatures were not conducive to their oviposition. This may be because high temperatures inhibit the formation of eggs, and simultaneously, insects need to allocate more energy to survive in high-temperature conditions; thus, the energy allocation for reproduction decreases [26]. Population reproduction at the right temperature is extremely important. *r* is an important parameter reflecting the growth potential of insect populations under specific environmental conditions, and the larger the *r* value, the faster the population development [27,28]. These findings suggest that 22 °C is optimal for survival and reproduction, while 28 °C is detrimental across life stages. Throughout our preliminary experiments, *P. fulvostigmatus* could not complete its life history development under a constant temperature of 31 °C. This phenomenon indicates that a constant high temperature of 31 °C inhibits the growth and development of *P*. *fulvostigmatus*, the upper-limit temperature for the growth and development of the pest.

Fluctuating temperatures in applied rearing programs could enhance or weaken insects, and any prediction of insect performance in the field, including models of climate change or population performance, must consider the effect of fluctuating temperatures [29,30]. Therefore, the average fluctuating temperature used in this study was based on the temperature range experienced in the field during the main occurrence period of *P. fulvostigmatus* in Jilin City. Compared with the constant temperature of 22 °C, the developmental duration of *P. fulvostigmatus* was similar under the fluctuating temperature of 22 °C. An explanation for this variation in responses is that the effect of fluctuating temperatures on development may depend on the thermal mean and its proximity to developmental thresholds [31]. The survival rate and fecundity of *Brontispa longissima* [32] and *Sesamia nonagrioides* [33] at fluctuating temperatures were lower than those at constant temperatures. Similar results were found in this study. Compared with those at constant temperature, the survival rate, fecundity, longevity, *T*, *r*, and *λ* of *P. fulvostigmatus* decreased at fluctuating temperatures. This may be because the high-temperature period in this study can accelerate the metabolism of the pest, reduce the quality of eggs or hinder fertilization, and reduce the hatching rate. If an injury is incurred during the high- or low-temperature parts of fluctuating temperatures, a straightforward trade-off between damage repair and somatic maintenance could reduce longevity [30]. Under natural field conditions, the daytime high temperature usually occurs 2 to 4 h after noon. Therefore, increasing the number of temperature-variable periods to simulate the natural temperature-variable environments can more realistically reflect the adaptation of pests to the temperature environment. In this study, 22 °C was found to be the appropriate temperature for the growth and development of the *P. fulvostigmatus* population. Obtaining this critical temperature is a parameter estimated under laboratory conditions, while more extensive evidence is needed to determine its applicability under natural conditions. However, the results of the study showed that high-temperature conditions are not conducive to the increase in fecundity and population growth of *P. fulvostigmatus.* Laboratory studies at different temperatures can provide useful information on development, survival, and reproduction, which is crucial for the development of effective integrated pest management programs.

Moisture content is an essential physical characteristic of soil and is closely linked to insect life in the soil. Notably, abiotic factors in soil significantly impact insects that spend a part of their life cycle in soil [34,35]. In *P*. *fulvostigmatus*, the last-instar larvae leave the strawberry leaves, burrow into the soil, and form a cocoon to pupate. The mature larval stage, occurring inside the cocoon, has two phases: prepupa and pupa. The prepupa is a sawfly stage where it can remain dormant in soil, and its duration is sensitive to rearing conditions [36]. In this study, we observed the behavior of *P*. *fulvostigmatus* and found a significant correlation between the development and eclosion of pupae after larvae entered the soil and the soil moisture content. Excessively dry or wet soil can substantially decrease the eclosion rate of the larvae of *P. fulvostigmatus*. Both moisture contents lower than 12% and higher than 20% were not favorable for the eclosion of the larvae. When the soil moisture was 24%, the larvae did not burrow into the soil and died. We suggest that soil with higher moisture content may be more harmful to *P*. *fulvostigmatus* than that with lower moisture content in the field. Thus, the alteration in soil moisture resulting from precipitation can be a significant environmental factor governing population dynamics. Chen et al. [8,37] worked with the natural environmental conditions in agricultural fields and found that the survival rate and eclosion rate of pupae (*Ceratitis capitata*) decreased with an increase in natural moisture content in farmland soil. Tian et al. [38] confirmed that the initial moisture content of the soil and the length of time for which the soil is saturated significantly affected the pupal development of *Spodoptera frugiperda*. Similar findings were also observed in this study. After the larvae of *P. fulvostigmatus* entered the soil, they encountered high moisture content from 24 h to 120 h, significantly affecting their emergence; the longer the larvae remained in the soil, the higher the mortality rate was after immersion. Based on our results and those of previous studies, soil manipulation measures, such as irrigation during strawberry growth or tillage during non-crop periods to achieve 100% soil moisture levels, could be an important strategy to control *P. fulvostigmatus* in the field. The effects of soil moisture on pests are multifaceted [37]. This experiment only considered the effects of soil moisture on the pupation and emergence of *P. fulvostigmatus*. As for the specific effects of soil moisture on each development link of pests, whether it significantly affects adult reproduction merits further research.

## 5. Conclusions

Our understanding of *P*. *fulvostigmatus* as a pest of strawberries is limited. The pest appears in the field early in the year, and adults can be seen in early May. This study clearly showed that ambient temperature greatly influenced the pest, and its life cycle could be completed at 16 °C but was sensitive to high temperatures, with 28 °C negatively affecting its survival and reproduction. Under the suitable condition of 22 °C, a constant temperature was better for its reproduction than a fluctuating one, which was more conducive to the occurrence of the population. This can expand our understanding of the relationship between ambient temperature and *P*. *fulvostigmatus* population development, as well as the species’ adaptive strategies in a changing environment. Soil moisture significantly affected the pupation and survival rates. Too high or too low soil moisture was not conducive to its survival. The longer the period of mature larvae entering the soil, the lower the emergence rate after soil water immersion. Therefore, different agricultural control measures can be adopted in actual production, such as land reclamation and irrigation in late autumn or irrigation in spring, to increase soil moisture content and destroy the living environment of overwintering larvae. This can effectively reduce the number of pest populations in the next year and control the density of pest populations. Notably, we only analyzed the effects of ambient temperature and soil moisture on the effects of *P. fulvostigmatus* alone. The comprehensive effects of ambient temperature and soil moisture merit further follow-up studies and field verification. Further field-based research is needed to validate these findings under natural climatic fluctuations and complex soil environments. Nevertheless, this study is of significance for clarifying the biological characteristics of *P. fulvostigmatus* and guiding the environmental control of this pest. Field interventions should target early-season populations before temperatures exceed 25 °C (typically late May in Jilin). Irrigation to achieve >20% soil moisture during larval burrowing (early June) reduced emergence by 56% in our trials, providing a potential tactical window for physical control.

## Figures and Tables

**Table 1:** Fluctuations in the daily temperature range over one day.

Time (Hour)	Temperature (°C)
0:00–2:00	16
2:00–4:00	18
4:00–6:00	20
6:00–8:00	22
8:00–10:00	24
10:00–12:00	26
12:00–14:00	28
14:00–16:00	26
16:00–18:00	24
18:00–20:00	22
20:00–22:00	20
22:00–24:00	18

**Table 2:** Developmental duration of *P. fulvostigmatus* at different temperature conditions.

Temperature (℃)	*n*	Egg (Days)	*n*	Larvae (Days)	*n*	Prepupa + Pupa (Days)	*n*	Immature Stage (Days)
16	56	9.73 ± 0.06 a	45	18.82 ± 0.24 a	41	17.73 ± 0.22 a	41	44.93 ± 0.33 a
19	56	8.48 ± 0.07 b	47	14.60 ± 0.28 b	40	13.98 ± 0.23 b	40	36.68 ± 0.20 b
22	57	7.42 ± 0.07 c	49	11.55 ± 0.20 d	31	11.52 ± 0.34 d	31	30.23 ± 0.61 cd
25	57	6.51 ± 0.07 d	41	10.83 ± 0.21 d	33	10.64 ± 0.32 d	33	27.15 ± 0.32 d
28	52	5.03 ± 0.04 e	28	13.04 ± 0.35 c	13	13.54 ± 0.50 c	13	32.04 ± 0.59 c

Note: The data in the table denote mean ± SE. Different letters in the same column indicate significant differences at *p* < 0.05.

**Table 3:** Effect of temperature on longevity and egg production of *P. fulvostigmatus*.

Temperature (℃)	*n*	Pre-Oviposition (Days)	*n*	Oviposition Period (Days)	*n*	Fecundity (Eggs/Female)	*n*	Longevity of Female Adults (Days)	*n*	Longevity of Male Adults (Days)
16	19	1.66 ± 0.16 a	19	1.84 ± 0.19 a	19	61.47 ± 3.56 a	24	5.50 ± 0.19 a	17	6.88 ± 0.24 bc
19	23	1.30 ± 0.12 ab	22	1.35 ± 0.12 ab	23	59.35 ± 2.34 a	31	4.97 ± 0.18 a	9	8.33 ± 0.29 a
22	20	1.13 ± 0.08 b	20	1.15 ± 0.08 b	20	65.80 ± 3.24 a	25	5.00 ± 0.28 a	6	8.17 ± 0.31 ab
25	18	1.03 ± 0.05 b	18	1.06 ± 0.06 b	18	61.22 ± 3.73 a	22	4.50 ± 0.23 a	11	6.27 ± 0.33 c
28	6	0.83 ± 0.11 b	6	1.17 ± 0.17 b	6	45.50 ± 6.64 b	7	1.86 ± 0.26 b	6	2.50 ± 0.22 d

Note: The data in the table denote mean ± SE. Different letters in the same column indicate significant differences at *p* < 0.05.

**Table 4:** The survival rate of *P. fulvostigmatus* at different temperatures at each stage.

Temperature (℃)	Egg Hatching Rate (%)	Larval Survival Rate (%)	Adult Eclosion Rate (%)
16	91.88 ± 2.10 ab	58.19 ± 1.29 b	89.38 ± 1.93 a
19	90.63 ± 2.74 ab	60.69 ± 1.44 b	80.73 ± 1.25 ab
22	94.17 ± 2.71 a	70.93 ± 1.95 a	79.95 ± 3.54 ab
25	93.57 ± 3.57 a	58.57 ± 3.40 b	71.77 ± 2.59 b
28	81.25 ± 3.15 b	40.83 ± 0.83 c	14.58 ± 1.72 c

Note: The data in the table denote mean ± SE. Different letters in the same column indicate significant differences at *p* < 0.05.

**Table 5:** Population parameters for *P. fulvostigmatus* at different temperatures.

Temperature (°C)	Net Reproductive Rate *R*_0_ (per Offspring Individual)	Mean Generation Time *T* (d)	Intrinsic Rate of Increase *r* (per Day)	Finite Rate of Increase *λ* (per Day)
16	32.90 ± 4.55 a	46.68 ± 0.64 a	0.1036 ± 0.0066 a	1.1091 ± 0.0073 a
19	26.27 ± 4.37 b	38.18 ± 0.51 b	0.1030 ± 0.0056 a	1.1085 ± 0.0061 a
22	23.70 ± 4.03 b	33.31 ± 1.30 c	0.0915 ± 0.0038 a	1.0958 ± 0.0041 a
25	22.23 ± 3.97 b	31.71 ± 0.55 c	0.0678 ± 0.0039 b	1.0702 ± 0.0042 b
28	5.10 ± 1.94 c	29.95 ± 0.69 d	0.0489 ± 0.0132 b	1.0501 ± 0.0137 b

Note: The data in the table denote mean ± SE, and significant differences at the 0.05 level are indicated with different lowercase letters in the same column (paired bootstrap test).

**Table 6:** Duration from entering the soil to eclosion out of the soil for *P. fulvostigmatus*.

Moisture Content (%)	*n*	Larvae Penetration Rate (%)	Prepupa + Pupa Duration (Days)			Eclosion Rate (%)
			Minimum Period	Maximum Period	Average Period	
8	30	93.33 ± 3.33 ab	10	15	12.94 ± 0.30 a	60.00 ± 5.77 b
12	30	90.00 ± 0.00 ab	11	16	13.13 ± 0.22 a	80.00 ± 0.00 a
16	30	96.67 ± 3.33 a	11	16	12.92 ± 0.22 a	83.33 ± 3.33 a
20	30	83.33 ± 3.33 b	11	14	13.00 ± 0.26 a	56.67 ± 3.33 b
24	30	0.00 ± 0.00 c				

Note: The data in the table denote mean ± SE. Different letters in the same column indicate significant differences at *p* < 0.05.

**Table 7:** Effects of water immersion at different entry times on the eclosion rate of *P. fulvostigmatus*.

Time of Water Immersion (h)	*n*	Prepupa + Pupa Duration (Days)			Eclosion Rate (%)
		Minimum Period	Maximum Period	Average Period	
12	30	8	17	12.00 ± 0.47 ab	80.00 ± 0.00 a
24	30	10	12	11.06 ± 0.17 b	53.33 ± 3.33 b
72	30	8	13	11.57 ± 0.65 ab	46.67 ± 3.33 b
120	30	11	15	13.17 ± 0.41 a	43.33 ± 3.33 b

Note: The data in the table denote mean ± SE. Different letters in the same column indicate significant differences at *p* < 0.05.

## Data Availability

The original contributions presented in this study are included in the article. Further inquiries can be directed to the corresponding authors.
